# Impact of Pemafibrate Therapy on Reducing Small Dense Low-Density-Lipoprotein-Cholesterol Levels in Patients with Hypertriglyceridemia

**DOI:** 10.3390/jcm12216915

**Published:** 2023-11-03

**Authors:** Yuki Hida, Teruhiko Imamura, Koichiro Kinugawa

**Affiliations:** Second Department of Internal Medicine, University of Toyama, Toyama 930-0194, Japan

**Keywords:** dyslipidemia, cardiovascular disease, heart failure

## Abstract

Background: Small dense LDL-cholesterol is a recently discovered cardiovascular risk factor beyond LDL-cholesterol. Pemafibrate is a novel selective peroxisome proliferator-activated receptor-α modulator that reduces triglyceride levels. Given the significant association between triglycerides and small dense LDL-cholesterol levels, pemafibrate may reduce the levels of small dense LDL-cholesterol. Methods: Patients with hypertriglyceridemia who started pemafibrate therapy and continued it for >3 months between 2018 and 2022 were included in this retrospective study. The levels of small dense LDL-cholesterol, which was estimated using Sampson’s equation, consisting of the LDL-cholesterol and triglyceride levels, were compared between baseline and 3-month follow-up. Results: A total of 98 patients receiving pemafibrate therapy (median age: 63 years, 69 male) were eligible, including 33 patients (34%) who received concomitant statins. Small dense LDL-cholesterol levels decreased significantly during the course of 3-month pemafibrate therapy from 48.9 (IQR: 35.7, 57.9) mg/dL to 38.8 (IQR: 30.0, 45.1) mg/dL, regardless of the concomitant administration of statins (*p* < 0.001). The rate of cardiovascular events decreased significantly from the pre-treatment 1-year period to the treatment 1-year period (from 13 to 2 events, from 0.133 to 0.021 events per year, incidence rate ratio: 0.16, 95% confidence interval: 0.14–0.17, *p* < 0.001). Conclusions: Pemafibrate therapy may mitigate the concentrations of small dense LDL-cholesterol autonomously in patients manifesting hypertriglyceridemia within the authentic clinical milieu. The clinical importance of the diminishment in small dense LDL-cholesterol instigated via pemafibrate merits further scrutiny.

## 1. Background

Strong statin therapy can lower low-density lipoprotein (LDL)-cholesterol levels and reduce the risk of cardiovascular diseases [1,2]. Thus, statin therapy is an established pharmacological therapy in primary prevention for high-risk cohorts and in secondary prevention for patients with cardiovascular diseases. However, there are still many patients with recurrent cardiovascular diseases, such as coronary artery disease, despite a significant reduction in LDL-cholesterol levels with strong statin therapy [3]. Further interventions beyond statins are currently desired to prevent the onset/recurrence of cardiovascular diseases.

Small dense LDL-cholesterol has emerged as a new clinical marker that is increasingly being used in clinical risk assessment for both primary and secondary prevention measures [4]. A large-scale study demonstrated that small dense LDL-cholesterol levels were associated with an increased risk of coronary artery diseases, even in a cohort with LDL-cholesterol being maintained under 100 mg/dL [5]. LDL-cholesterol-lowering therapy may be insufficient to completely prevent the development of cardiovascular diseases, and small dense LDL-cholesterol may be the key to further preventing such comorbidities upon undergoing fundamental LDL-lowering statin therapy.

The total LDL-cholesterol is made up of large buoyant LDL-cholesterol and small dense LDL-cholesterol. Specifically, small dense LDL-cholesterol has a high specific gravity and comprises small particles [6]. The proportion of small dense LDL-cholesterol can be expected to increase in the presence of various comorbidities, including diabetes mellitus, obesity, insulin resistance, and hypertriglyceridemia. Small dense LDL-cholesterol may be strongly associated with the accelerated progression of atherosclerosis, owing to its greater adhesion to endothelial cells because of its small size and susceptibility to oxidation, in addition to the background multi-comorbidities that also progress atherosclerosis [7]. Collectively, it is postulated that small dense LDL-cholesterol constitutes a risk factor for the advancement of cardiovascular maladies, surpassing the influence of LDL-cholesterol levels in isolation [8]. How can we intervene in cases of elevated small dense LDL-cholesterol levels?

Pemafibrate is a recently introduced selective peroxisome proliferator-activated receptor alpha (PPARα) activator [9]. Pemafibrate can reduce triglyceride levels to the same extent as other conventional fibrates, with a lower incidence of drug-related adverse events [10,11,12]. As triglyceride levels are a primary determinant of LDL particle size and buoyancy, pemafibrate may theoretically decrease small dense LDL-cholesterol levels. A therapeutic reduction in small dense LDL-cholesterol may prevent the future development of cardiovascular diseases and improve patients’ mortality and morbidity [13]. Unfortunately, a randomized controlled trial, PROMINENT, failed to demonstrate any benefit of pemafibrate therapy in reducing cardiovascular events compared with a placebo. Nevertheless, the way in which pemafibrate reduced small dense LDL-cholesterol levels in this study was unknown, and further detailed knowledge about the association between pemafibrate therapy and small dense LDL-cholesterol levels should let us know the optimal therapeutic strategy to appropriately reduce small dense LDL-cholesterol levels using pemafibrate for a reduction in cardiovascular risks.

Unfortunately, small dense LDL-cholesterol levels cannot be routinely measured in most clinical laboratories, since they are not currently reimbursable. Recently, Sampson’s equation has been introduced to calculate small dense LDL-cholesterol levels via simply using several standard lipid parameters [14]. In this retrospective study, we evaluated the effect of pemafibrate on reducing small dense LDL-cholesterol levels, which was estimated using Sampson’s equation in patients with hypertriglyceridemia together with a variety of comorbidities in real-world clinical practice.

## 2. Methods

### 2.1. Patient Selection

Patients with hypertriglyceridemia who received pemafibrate for the first time and continued it for over 3 months at our institute between October 2018 and November 2022 were included in this retrospective study. Patients who initiated pemafibrate by switching from other fibrates were not included. Patients who initiated pemafibrate at other institute and continued it in our institute were not included. Patients were followed at our institute or affiliated institutions for 1 year or until Jun 2023. Other medications were titrated during pemafibrate therapy at the discretion of the attending physicians. Duly executed informed consents were solicited from all participants prior to their inclusion in the study. The study protocol received approval from the institutional review board under the reference R2015154 on 11 April 2016.

### 2.2. Biomarker Measurement

The primary concern of this study was a trend in estimated small dense LDL-cholesterol levels during the 3-month therapeutic period. Laboratory data, encompassing lipid parameters, were subjected to conventional laboratory techniques. Serum and plasma samples were collected at baseline, just prior to the commencement of pemafibrate therapy, and again at the three-month follow-up point, all under fasting conditions, and were promptly frozen at −80 degrees Celsius.

The small dense LDL-cholesterol level was calculated from other common lipid parameters via the previously proposed Sampson’s equation: [14] (small dense LDL-cholesterol) = (LDL-cholesterol) − (large buoyant LDL-cholesterol). Here, (large buoyant LDL-cholesterol) = 1.43 × (LDL-cholesterol) − [0.14 × [ln (triglyceride)] × (LDL-cholesterol)] − 8.99. For example, when a patient had 143 mg/dL of LDL-cholesterol and 328 mg/dL of triglyceride, his/her small dense LDL-cholesterol was calculated as 63.5 mg/dL.

### 2.3. Other Clinical Data

Demographics, comorbidities, and medication data were obtained just before pemafibrate initiation as baseline characteristics. Data regarding concomitantly administered statins were retrieved. Laboratory data, including lipid profiles, were obtained at baseline and 3 months later. Laboratory data were followed routinely at scheduled out-patient clinics, irrespective of patients’ complaints, including hemoglobin, liver enzymes, renal parameters, blood glucose, and creatinine kinase. We used a revised equation for the estimated glomerular filtration rate from serum creatinine levels in the Japanese cohort [15].

Cardiovascular events, including heart failure, coronary artery disease, and stroke, were counted for 1 year pre-treatment and 1 year of treatment.

### 2.4. Statistical Analysis

Continuous variables were reported in the form of medians (lower quartile, upper quartile), a practice that applies them uniformly, irrespective of their distribution, owing to the relatively moderate sample size. Categorical variables were expressed as numerical counts and corresponding percentages. Small dense LDL-cholesterol levels were calculated according to the previously proposed Sampson’s equation, as detailed above. The trends in lipid parameters, including estimated small dense LDL-cholesterol, were assessed using the Wilcoxon signed-rank test. The rate of cardiovascular events was compared between 1 year pre-treatment and 1 year of treatment using negative binomial regression analysis. A level of significance denoted by *p* < 0.05 was considered statistically meaningful. The statistical analyses were conducted using SPSS Statistics 23, a product of SPSS Inc., located in Armonk, IL, USA.

## 3. Results

### 3.1. Baseline Characteristics

A total of 116 patients were considered for inclusion. Of these, 18 patients who had already started pemafibrate at a former institute or had converted pemafibrate from other fibrates were excluded. Finally, 98 patients who received pemafibrate on a de novo basis to treat their dyslipidemia were included in this retrospective study (Table 1). The median age was 63 (53, 71) years, and 69 (70%) were male patients. Half of the patients (51%) had diabetes mellitus. No patients had received other fibrates beforehand. Thirty-three patients (34%) received statins concomitantly.

### 3.2. Trend in Small Dense LDL-Cholesterol

**Figure 1:**
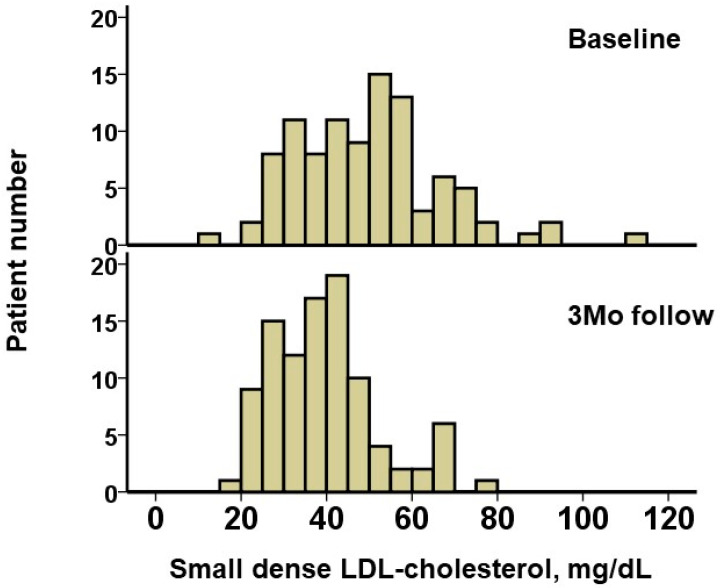
Distribution of small dense LDL-cholesterol levels at baseline and 3 months after the initiation of pemafibrate. LDL, low-density lipoprotein.

The baseline triglyceride level was 310 (238, 489) mg/dL, and baseline HDL-cholesterol level was 45 (36, 54) mg/dL (Table 2). The small dense LDL-cholesterol was estimated as 48.9 (35.7, 57.9) mg/dL. A distribution of small dense LDL-cholesterol is displayed in Figure 1.

**Figure 2:**
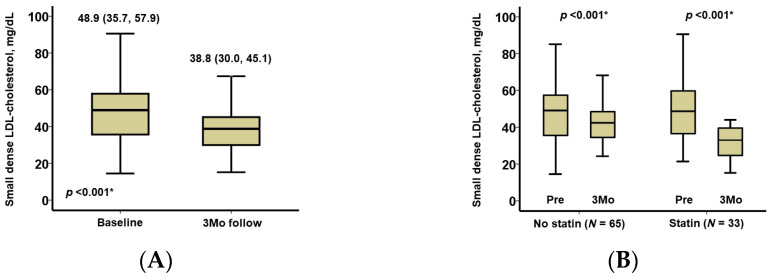
Trends in small dense LDL-cholesterol over 3 months of pemafibrate therapy in all cohorts (**A**) and in the sub-groups with and without statins (**B**). LDL, low-density lipoprotein. Trends were assessed using the Wilcoxon signed-rank test. * *p* < 0.05. Small dense LDL-cholesterol levels decreased significantly over 3 months of pemafibrate therapy, regardless of the concomitant administration of statins.

During a median follow-up period of 365 (365, 365) days, no patients had drug-related adverse events such as hepatic injury and no patients terminated pemafibrate. After 3 months of pemafibrate treatment, triglyceride levels decreased significantly down to 148 (114, 253) mg/dL, and HDL-cholesterol increased significantly up to 49 (40, 59) mg/dL (*p* < 0.001 for both; Table 2). LDL-cholesterol remained unchanged (*p* = 0.91). The distribution of small dense LDL-cholesterol at the 3-month follow-up is displayed in Figure 1. The level of small dense LDL-cholesterol decreased significantly, down to 38.8 (30.0, 45.1) mg/dL (*p* < 0.001; Figure 2A).

Thirty-three patients received statins concomitantly. Small dense LDL-cholesterol levels decreased significantly after 3 months of pemafibrate therapy, regardless of the administration of statins (*p* < 0.001 for both; Figure 2B). LDL-cholesterol levels were significantly lower in patients receiving statins (88 [70, 107] versus 119 [95, 137] mg/dL, *p* < 0.001).

### 3.3. Association between Change in Small Dense LDL-Cholesterol and Other Lipid Parameters

**Figure 3:**
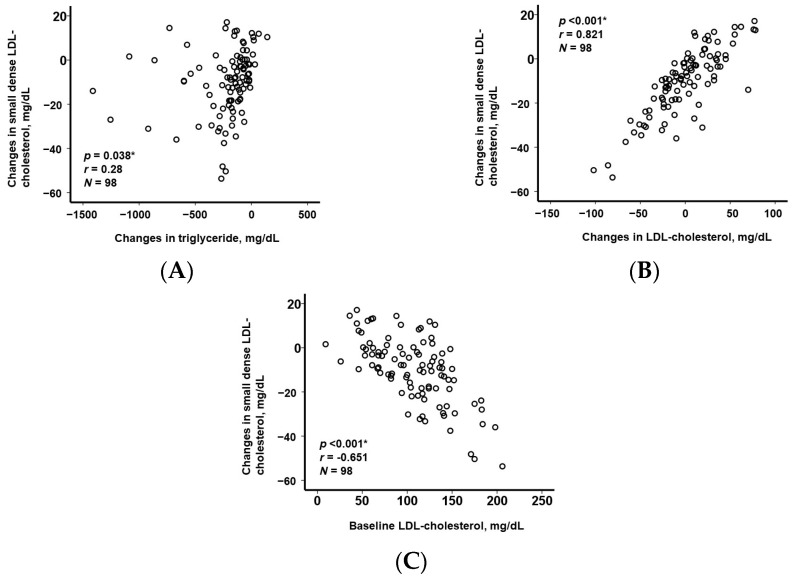
The correlation between the change in small dense LDL-cholesterol and the change in triglyceride (**A**); the change in LDL-cholesterol (**B**); and the baseline LDL-cholesterol (**C**). * *p* < 0.05 via Pearson’s correlation coefficient. Both a greater decrease in triglyceride and a greater decrease in LDL-cholesterol were correlated with a greater decrease in small dense LDL-cholesterol, respectively. A lower baseline LDL-cholesterol was associated with a lesser change in small dense LDL-cholesterol.

A greater decrease both in triglyceride and in LDL-cholesterol was correlated with greater decreases in small dense LDL-cholesterol (*p* < 0.05 for both; Figure 3A,B). A baseline lower LDL-cholesterol was correlated with a lesser decrease in small dense LDL-cholesterol (*p* < 0.001, r = −0.651 Figure 3C).

### 3.4. Cardiovascular Events

**Figure 4:**
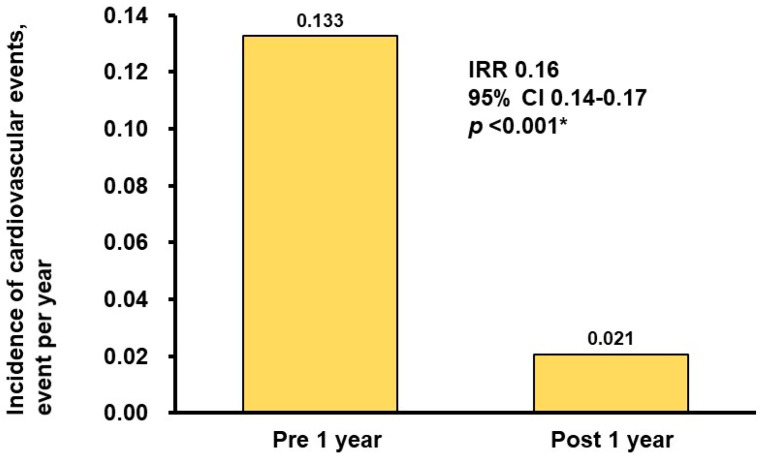
Comparison of the event rate between the 1-year pre-treatment period and the 1-year treatment period. IRR, incidence rate ratio; CI, confidence interval. Event rates were compared between the two groups using negative binomial regression analysis. The event rate was expressed as the event number per year. * *p* < 0.05. The rate of cardiovascular events decreased significantly after 1 year of pemafibrate therapy compared with a pre-treatment period.

During a 1-year observation period prior to the initiation of pemafibrate, the rate of cardiovascular events was 0.133 per year (Figure 4). During the 1-year treatment period, all patients continued pemafibrate without any drug-related adverse events. The rate of cardiovascular events was 0.021 per year, which was significantly lower than that in the pre-treatment period (incidence rate ratio: 0.16, 95% confidence interval: 0.14–0.17, *p* < 0.001). During pemafibrate therapy, one patient was on statins, and another was not.

### 3.5. Post Hoc Power Analysis

We performed a power analysis for the primary concern: the trend in small-dense LDL cholesterol after the administration of pemafibrate. The alpha error was defined as 0.05, the total sample size was 98, and an effect size was calculated as 0.65. A 1-beta value was calculated as 0.99.

## 4. Discussion

In this retrospective investigation, we conducted an assessment of the trajectory of small, dense LDL-cholesterol concentrations, as ascertained through the application of the recently proposed Sampson’s equation, throughout a 3-month course of pemafibrate therapy. Over the course of this three-month pemafibrate intervention, the estimated levels of small, dense LDL-cholesterol exhibited a noteworthy and statistically substantial reduction, irrespective of concurrent statin administration.

During the one-year span of pemafibrate treatment, all patients consistently adhered to the pemafibrate regimen, with no documented occurrences of drug-related adverse events. Furthermore, when comparing the one-year period of pemafibrate therapy to the one-year pretreatment interval in the absence of pemafibrate, a significantly diminished incidence of cardiovascular events was observed.

### 4.1. Pemafibrate and Small Dense LDL-Cholesterol

Pemafibrate is a recently innovated selective peroxisome proliferator-activated receptor alpha (PPARα) activator. Pemafibrate appears to offer a superior alternative to traditional fibrates, including fenofibrate, for the amelioration of hypertriglyceridemia, all the while exhibiting a relatively reduced incidence of drug-related adverse events, notably alleviating concerns related to hepatic failure, a well-recognized limitation of traditional fibrate therapy. The safety and efficacy of pemafibrate have been substantiated through extensive investigations involving sizable cohorts representing diverse medical conditions, thoughtfully curated for the purpose of rigorous examination [10,11,12]. In phase II and phase III clinical trials, pemafibrate decreased triglyceride levels greater than conventional fenofibrate. The impact of pemafibrate on reducing triglyceride levels was confirmed also in patients receiving statins in another phase III trial [16].

Triglyceride levels are closely associated with the presence of small dense LDL-cholesterol [17]. The number of small dense LDL-cholesterol particles may theoretically increase along with triglyceride levels, particularly when the level of LDL-cholesterol is high. In the clinical literature, there is a mild-to-moderate correlation between triglyceride levels and actual measured small dense LDL-cholesterol levels [18]. Thus, our finding that pemafibrate decreased small dense LDL-cholesterol levels along with triglyceride levels may not be surprising, although it had not been previously validated [19]. A very recent study also showed that the addition of pemafibrate to statins was superior to doubling a statin dose when it came to reducing small dense LDL-cholesterol levels, irrespective of the statin type, in carefully selected patients with type 2 diabetes and hypertriglyceridemia [20].

On the contrary, other variables are also associated with a higher burden of small dense LDL-cholesterol. For example, insulin resistance, which is often seen in patients with metabolic syndrome, can also increase the burden of small dense LDL-cholesterol [21], especially in patients with obesity [18]. A recent study found that pemafibrate improved insulin resistance and maintained beta-cell function, which were correlated with improvement in lipid abnormality [22].

Considering these various potential confounders, we used Sampson’s equation to estimate small dense LDL-cholesterol levels, instead of using other surrogate markers [13]. This equation has been validated in patients with a variety of diseases [23]. A recent study using health checkup mega-data also calculated small dense LDL-cholesterol levels using this equation, and the calculated small dense LDL-cholesterol predicted the future incidence of diabetes [24]. In particular, we should understand that the estimated small dense LDL-cholesterol can be overestimated in patients with low lipid profiles, for example, during pemafibrate treatment like in our study. In other words, the impact of any interventions to lower the small dense LDL-cholesterol levels can be underestimated when we use Sampson’s equation. Nevertheless, we demonstrated for the first time that the estimated small dense LDL-cholesterol levels decreased significantly after 3 months of pemafibrate therapy in this study.

### 4.2. Impact of Pemafibrate on Reducing Cardiovascular Events

The effect of pemafibrate on reducing adverse events remains controversial. A large-scale randomized control trial, PROMINENT, failed to demonstrate the benefit of pemafibrate therapy in reducing cardiovascular events compared with a placebo [13]. In this trial, LDL-cholesterol levels were tightly controlled with statins to less than 70 mg/dL. In patients with extremely low LDL-cholesterol, small dense LDL-cholesterol should also be low, and the effect of pemafibrate on reducing small dense LDL-cholesterol may be limited.

LDL-cholesterol levels were more mildly controlled around 100 mg/dL in our study. Also, in our study, patients with lower baseline LDL-cholesterol could rarely achieve a greater decrease in small dense LDL-cholesterol levels during pemafibrate therapy. Nevertheless, given that the effect size was calculated as 0.65, the reduction in small dense LDL-cholesterol during pemafibrate therapy would be significant in the total cohort. The current study is a proof of concept, and further studies are warranted to clarify the impact of pemafibrate on reducing small dense LDL-cholesterol in a variety of clinical scenarios.

The therapeutic target level for small dense LDL-cholesterol is another concern. A previous study suggested a cutoff of 20.9 mg/dL for secondary prevention in patients with acute coronary syndrome, [25] while another study proposed a cutoff of 32.6 mg/dL in a similar cohort [26]. The median value of small dense LDL-cholesterol in our study was 38.8 mg/dL. Few studies has evaluated the target for small dense LDL-cholesterol for primary prevention. A recent study involving individuals receiving an annual health checkup showed a cutoff of 42 mg/dL to predict the development of de novo ischemic heart disease [27]. A lower target for small dense LDL-cholesterol may be needed for secondary prevention than for primary prevention [28]. Again, this study is a proof of concept. We demonstrated that pemafibrate could reduce the level of small dense LDL-cholesterol, but its optimal therapeutic target during pemafibrate therapy, as well as optimal patient selection, should be investigated in the next study. For example, patients intolerant to aggressive statin therapy or those with advanced cardiovascular diseases may be good candidates for aggressive pemafibrate therapy via reducing small dense LDL-cholesterol levels.

### 4.3. Limitations

Our investigation was conducted with a limited cohort size and a relatively brief observational duration. It is imperative to underscore the significance of a protracted follow-up period to comprehensively assess the prognostic implications of pemafibrate within an authentic clinical setting. In light of the restricted sample size, we treated all continuous variables as non-parametric data. Non-significance in this study does not indicate similarity. Given the retrospective nature of this study, it has limitations in terms of poor control over the exposure factors, covariates, and potential confounders. To minimize these limitations, we attempted to collect comprehensive data. We included only de novo cases. This is a proof-of-concept study, and further, larger-scale studies are warranted to validate our findings. Notably, we should investigate the optimal target level of small dense LDL-cholesterol during pemafibrate therapy to obtain greater clinical outcomes. We lacked a control group and compared the clinical parameters between baseline and three months later. Given the established evidence of pemafibrate therapy on hypertriglyceridemia, it may be ethically prohibited to create a control group that do not receive any intervention to their hypertriglyceridemia. The measurement of small dense LDL-cholesterol has not yet been approved by insurance so far, and we used the formula to calculate the amounts. Thus, we believe that most other institutions, which cannot measure small dense LDL-cholesterol either, can use our findings. The indication of pemafibrate was at the discretion of the attending physicians. They may have decided to indicate pemafibrate to patients with advanced cardiovascular diseases or those at high risk of cardiovascular diseases, which may constitute a selection bias.

## 5. Conclusions

Pemafibrate, a recently introduced discerning activator of PPARα, has exhibited a notable capacity to mitigate levels of small, dense LDL-cholesterol, as assessed through the contemporary Sampson’s equation. The clinical implications stemming from this reduction in small, dense LDL-cholesterol levels, as well as the precise criteria for patient selection suitable for pemafibrate therapy, and the establishment of therapeutic thresholds for small, dense LDL-cholesterol levels, are matters of paramount concern. These topics merit rigorous validation through more expansive prospective studies.

## Figures and Tables

**Table 1:** Baseline characteristics.

	N = 98
Age, years	63 (53, 71)
Male sex	69 (70%)
Statin use	33 (34%)
Hypertension	69 (71%)
Atrial fibrillation	11 (11%)
Diabetes mellitus	49 (51%)

Continuous variables are stated as median and interquartile, and categorical variables are stated as numbers and percentages.

**Table 2:** Trend in laboratory parameters.

	Baseline (N = 98)	3Mo Follow-Up (N = 98)	*p* Value
Hemoglobin, g/dL	14.2 (12.9, 15.4)	13.5 (12.7, 15.2)	0.22
eGFR, mL/min/1.73 m^2^	70.5 (60.3, 80.6)	65.0 (44.1, 81.6)	0.75
Asparate aminotransgerase, IU/L	25 (12, 36)	23 (14, 39)	0.43
Alanine aminotransgerase, IU/L	26 (12, 43)	24 (13, 44)	0.54
Blood glucose, mg/dL	97 (73, 108)	95 (74, 106)	0.76
Creatine kinase, IU/L	143 (84, 198)	153 (94, 221)	0.26
TG, mg/dL	310 (238, 489)	148 (114, 253)	<0.001 *
HDL-cholesterol, mg/dL	45 (36, 54)	49 (40, 59)	<0.001 *
LDL-cholesterol, mg/dL	111 (75, 136)	104 (81, 136)	0.91
Total cholesterol, mg/dL	208 (178, 242)	172 (151, 211)	<0.001 *
TG-rich lipoprotein, mg/dL	48 (35, 70)	26 (21, 45)	<0.001 *

Continuous variables are stated as median and interquartile. Laboratory parameters were obtained at baseline just before the initiation of pemafibrate and at 3 months after the initiation of pemafibrate. * *p* < 0.05 via Wilcoxon signed-rank test. eGFR, estimated glomerular filtration rate; TG, triglyceride; HDL, high-density lipoprotein; LDL, low-density lipoprotein.

## Data Availability

Data, including the study protocol, can be made accessible to interested parties through correspondence with the designated corresponding authors upon reasonable inquiry.
